# A review of progress towards sub-national malaria elimination in Matabeleland South Province, Zimbabwe (2011–2015): a qualitative study

**DOI:** 10.1186/s12936-018-2299-0

**Published:** 2018-04-03

**Authors:** Gladwin Muchena, Busisani Dube, Rudo Chikodzore, Jasper Pasipamire, Sivakumaran Murugasampillay, Joseph Mberikunashe

**Affiliations:** 1 Ministry of Health and Child Care, P. Bag A5225, Matabeleland South Province Bulawayo, Zimbabwe; 2 Ministry of Health and Child Care, National Malaria Control Programme, Harare, Zimbabwe; 3 World Health Organization, Zimbabwe Country Office, Harare, Zimbabwe; 4 World Health Organization, Global Malaria Programme, Geneva, Switzerland

**Keywords:** Malaria, Review, Performance, Elimination, Incidence

## Abstract

**Background:**

Malaria remains a public health problem in Zimbabwe. However, malaria elimination has become a foreseeable prospect with Matabeleland South Province making significant gains towards halting local malaria transmission. This study reviews malaria elimination progress and challenges to date utilizing the World Health Organization’s Malaria Programme Review framework.

**Results:**

Between 2011 and 2015, malaria incidence was less than one case per 1000 population at risk in all districts save for Beitbridge and Gwanda. The majority of cases were from Beitbridge with local transmission in the same. Incidence declined in Bulilima (p = 0.01), Gwanda (p = 0.72) and Umzingwane (p = 0.44), increasing in Beitbridge (p = 0.35), Insiza (p = 0.79) and Mangwe (p = 0.60). Overall provincial incidence declined although this was not statistically significant. Malaria transmission was bimodal, with a major peak in April and a minor peak in October. A case based malaria surveillance system existed but was not real-time. Foci response guidelines were not domesticated. Artemisinin formed the backbone of case management regimens with primaquine for gametocyte clearance. Indoor residual spraying coverages were below the national target of 95% for rooms targeted for spraying.

**Conclusion:**

Matabeleland South province has set precedence for targeting sub-national malaria elimination in Zimbabwe. This experience may prove useful for national scale up. There is need to improve surveillance, foci response and intensification of activities to halt residual malaria transmission in Beitbridge District.

## Background

Matabeleland South Province is one of the eight rural provinces in Zimbabwe. It lies on the southernmost side of Zimbabwe, bordering with South Africa along the Limpopo River and with Botswana to the west. The province has a population of about 683,893 [1]. In 2003, the Trans-Limpopo Malaria Initiative was launched, aiming to help reduce malaria burden in Southern Africa along the Limpopo River. Matabeleland South Province in 2012 reoriented its programme to carrying out malaria elimination activities in all of its seven districts [2].

The World Health Organization recommends Malaria Control Programme Performance Reviews as tools to improve the operational performance and strategic direction for “scaling up delivery of a mix of anti-malaria interventions in order to reduce malaria morbidity and mortality and overall transmission” [3]. Malaria reviews are an adaptation of public health performance management of malaria interventions [4]. This is very critical for the identification of major achievements, lessons learnt and best practice, critical issues, priority problems. Within the context of problem solving, this is a step towards addressing these and re-aligning the programme for better performance towards set targets and goal [5]. These reviews are carried out at the end of a Malaria Strategic Plan cycle and guide the development of the next strategic plan. Malaria Programme Performance Reviews typically include the following: Phase 1 Planning programme review; Phase 2 Internal thematic desk review; Phase 3 Joint programme field review; Phase 4. Final report and follow up of recommendations and updating policies, strategic and annual operational plans and programme re-design. These reviews are period and jointly carried by national, international bodies and partners [6]. The aim of this study was to assess progress made in Matabeleland South Province toward malaria elimination. Findings of the study would be used to identify challenges and inform future programming.

## Methods

An end term Malaria Programme Review (MPR) was conducted using a modified WHO Malaria Programme Reviews methodology. WHO Malaria Programme Reviews typically utilize standardized qualitative questionnaires which follow these themes: programme management, malaria diagnosis and case management, social and behaviour change communication, commodities procurement and supply chain management and vector control. Desk reviews, field data verification and key informant interviews in three Rural Health Centres, one District Hospital, one, Provincial Hospital, one District Health Executive and the Provincial Health Executive were carried out. The following facilities were conveniently enrolled into the study: Manama Mission Hospital, Beitbridge District Hospital, Gwanda Provincial Hospital, Chitulipasi, Chasvingo and Nhwali Rural Health Centres. Trend lines were fitted to malaria incidence across 5 years using linear regression at α = 0.05 to test for significance of change in incidence.

## Results

### Description of the study site

**Fig. 1:**
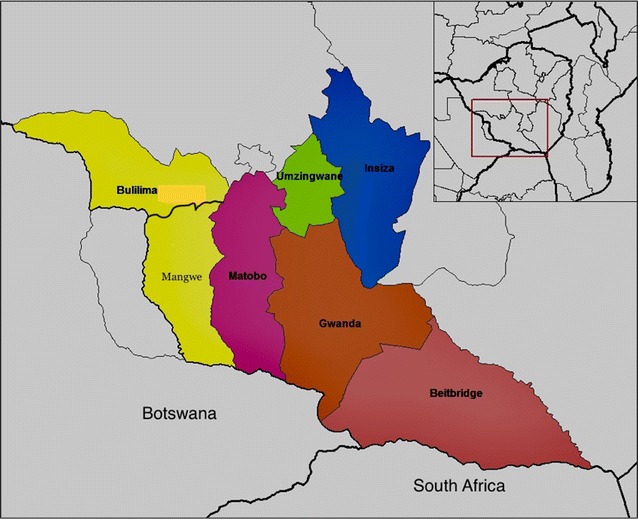
Map showing Matabeleland South Province districts. Available from: https://commons.wikimedia.org/wiki/File:Matabeleland_South_districts.png

Matabeleland South Province is located to the south of Zimbabwe, in Southern Africa. It has seven administrative districts, with Beitbridge being at the southernmost tip, bordering with South Africa (see Fig. 1).

### Malaria incidence in Matabeleland South Province

**Fig. 2:**
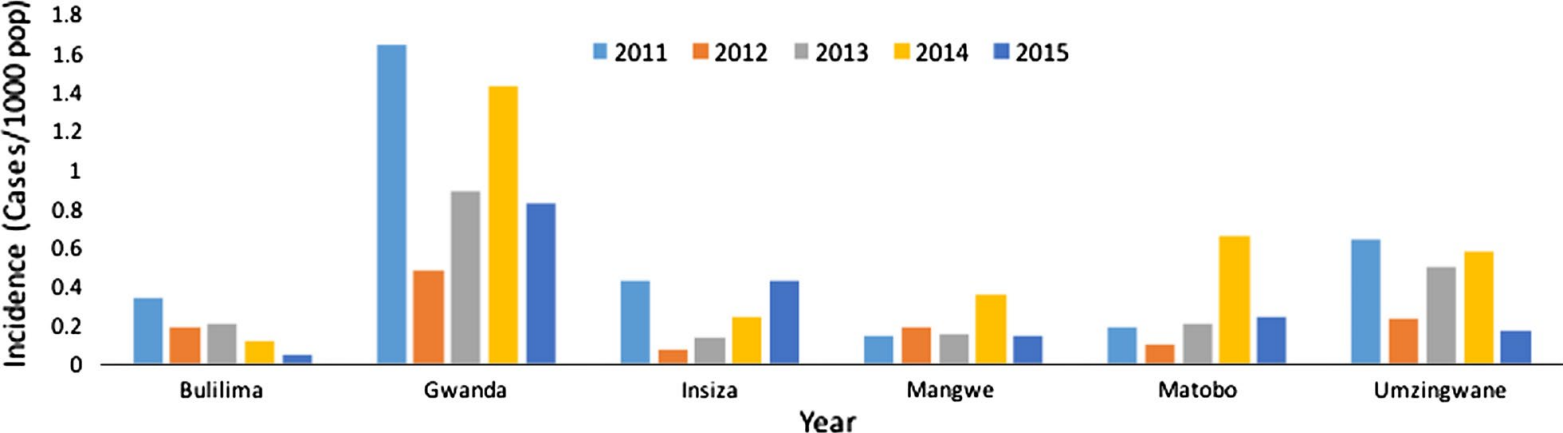
Malaria incidence Matabeleland South (without Beitbridge District), 2011–2015

**Fig. 3:**
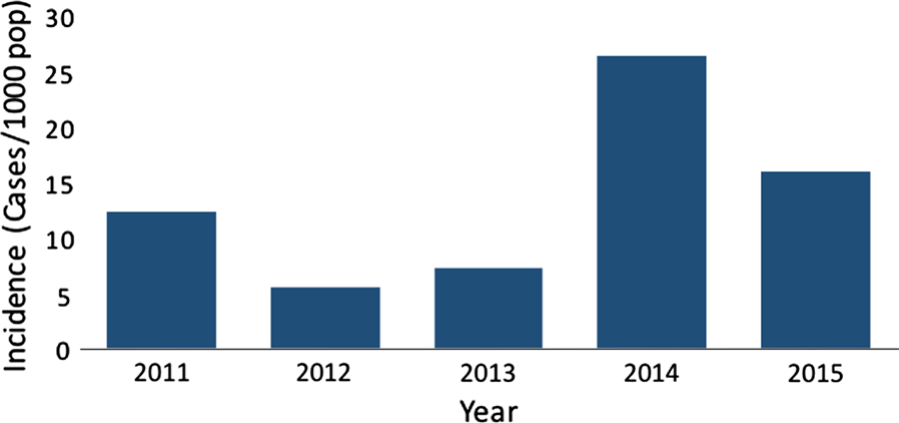
Malaria incidence Beitbridge District, 2011–2015

**Fig. 4:**
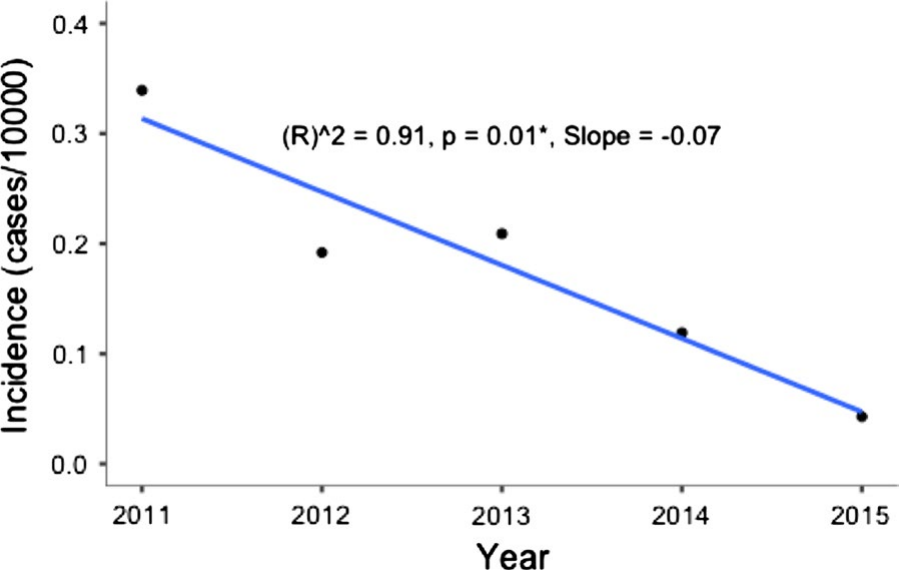
Malaria incidence trend Bulilima District, 2011–2015

**Fig. 5:**
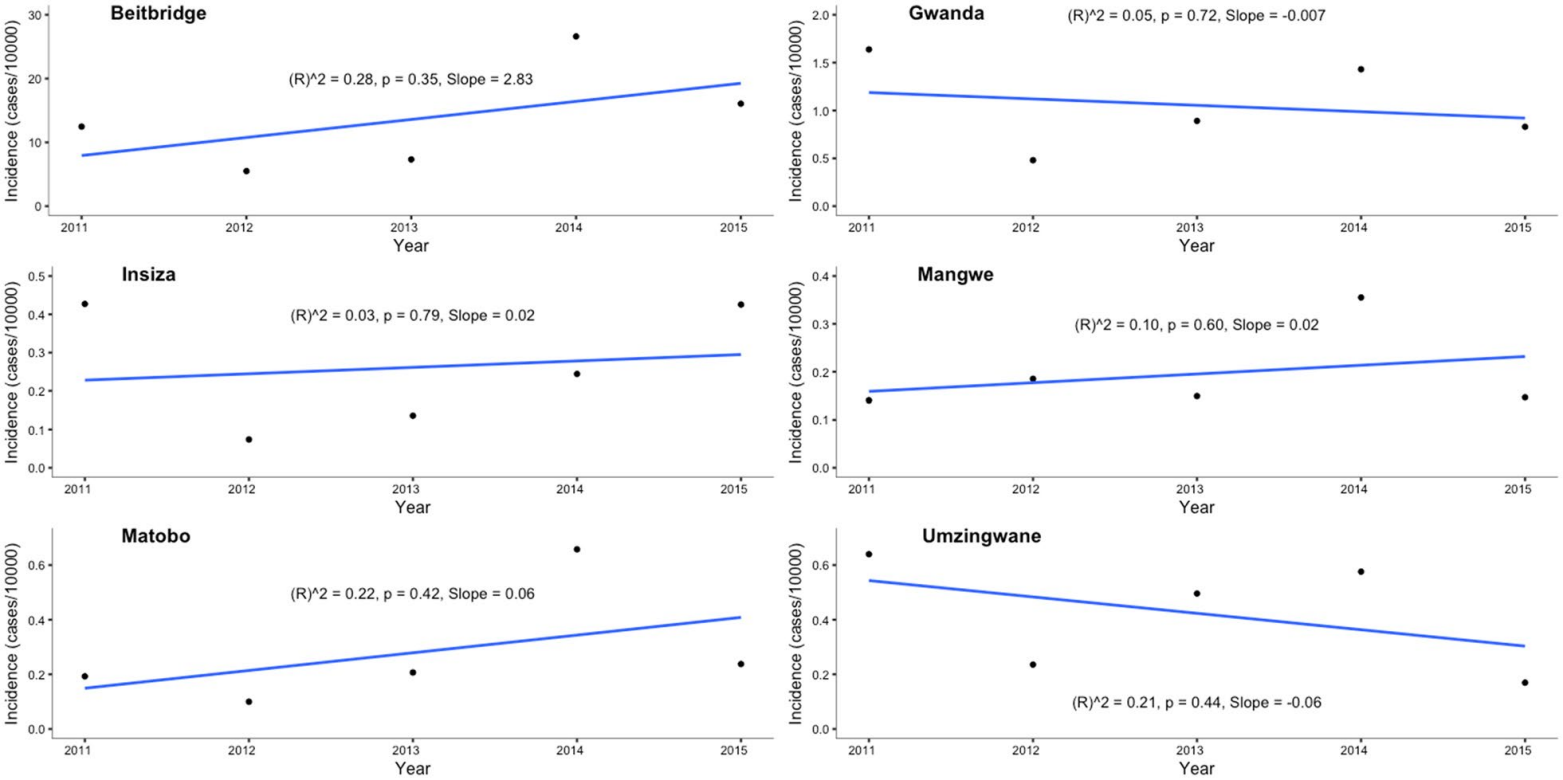
Malaria incidence trends for remaining districts, 2011–2015

Malaria incidence in five of the seven districts was below 1 case per 1000 population at risk from 2011 to 2015. In Gwanda District, the highest malaria incidence was 1.60 per 1000 population at risk, whilst Beitbridge District had the highest incidence of 26 per 1000 population at risk in 2014 (Fig. 2). The decline in incidence in Bulilima District was statistically significant (p = 0.01). Changes in incidence in all other districts were no statistically significant (Figs. 3, 4 and 5).

**Fig. 6:**
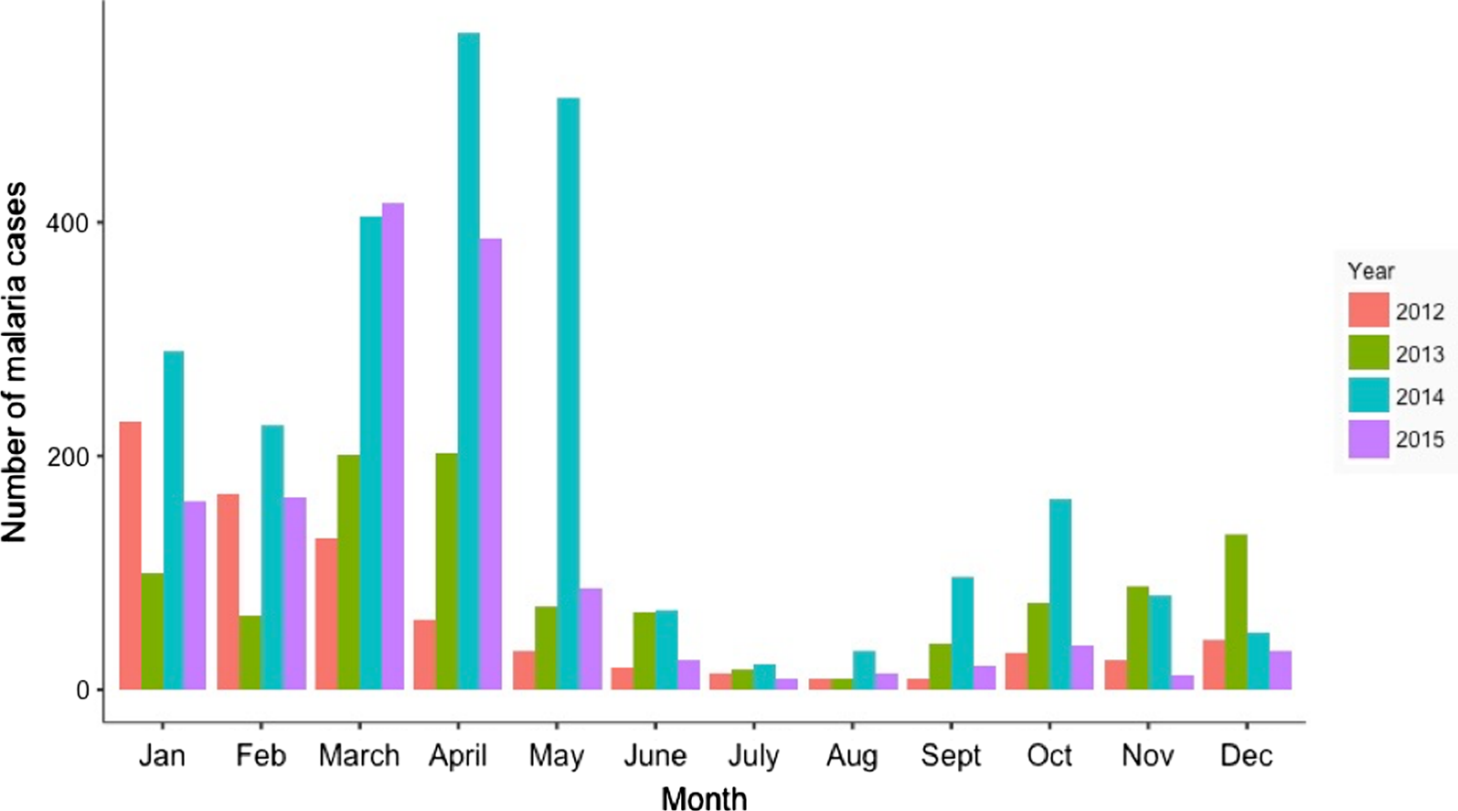
Seasonal malaria transmission in Matabeleland South Province

Malaria transmission in the province was bimodal and seasonal with peaks in April and October. The major peak was in April with the minor peak in October. Beitbridge, the highest burdened district mirrors the same pattern (Fig. 6). In the 2014–2015 season, cases imported into Matabeleland South were from South Africa (56%), Malawi (16%) and Mozambique (12%).

### Case-based surveillance for malaria

A case based malaria surveillance system was started in the third quarter of 2012 as a paper based system for case notification, case investigation and foci investigation on days 1, 3 and 7, respectively. This was upgraded in 2014 to an electronic system with the use of Personal Digital Assistants linked to a central server. This system operated on Windows Mobile Operating System. Environmental Health Technicians (EHTs) are trained in the use of the case base surveillance tools. Notification was done by a clinician at the time of diagnosis. The EHT was responsible for capturing data from paper to electronic format and sending it to a central server. The EHTs conduct reactive case detection screening of household contacts. There was identification, geo-coding and mapping of surrounding potential and active Anopheles breeding sites. Some of the breeding sites were treated with larvicide in Beitbridge in 2014. However, since 2015 there has been no larvicide available. In Beitbridge District there were delays in case investigation with incomplete case investigation done during the IRS season and none in the first 2 months of 2016. There was no real time notification of key staff upon case notification. The storage of elimination data was poor. Malaria registers were present but incompletely filled in. Foci registers were absent and there was no foci database at district and provincial levels.

### Malaria epidemic preparedness and response

The national weekly disease surveillance system was the cornerstone of Epidemic Preparedness and Response (EPR). Malaria cases at facility level were tallied onto T3 forms and summarized every week before submission to the district level. Malaria thresholds were sent out by the District Health Information Officer to all health facilities. Primary health facilities had malaria thresholds and a malaria spot map displayed. In 2014 there was an outbreak reported at Chitulipasi Clinic in Beitbridge District. Thresholds were calculated using the “mean plus 1.5 standard deviation” formula at Chitulipasi Rural Health Centre. Health facility and District EPR plans were present. The EPR plan in Gwanda District was costed. There were buffer stocks of rapid diagnostic test kits and malaria medicines in the two districts.

### Malaria programme information, monitoring and evaluation systems

The Health Information System included both outpatient and inpatient data. Indicators captured included number of malaria cases by age, artemisinin-based combination therapy (ACT) given, malaria deaths, malaria suspects seen, malaria suspects tested by either rapid diagnostic test (RDT) or microscopy. Data on IRS coverage was available. From the district level onwards, this data is available on a web based system, DHIS2. This is used to prepare monthly, quarterly and annual malaria programme monitoring and evaluation reports for district and provincial health teams programme and health system performance based management.

### Malaria operational research

In 2014, Therapeutic Efficacy Testing was carried out by the National Malaria Control Programme. However, other research was mostly carried out by collaborative bodies and not necessarily the Provincial Directorate itself. The Provincial Directorate did not have an operational research budget.

### Vector control

**Fig. 7:**
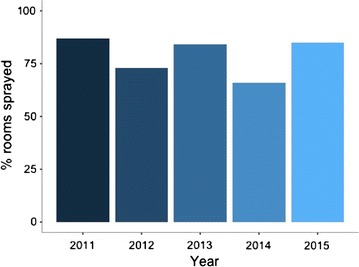
IRS coverage in Matabeleland South

The main malaria vector in Matabeleland South was *Anopheles gambiae* sensu lato (s.l.) and *Anopheles arabiensis. Anopheles quadriannulatus* was identified as a potential vector in the province. Susceptibility tests using the WHO Tube tests conducted in 2015 revealed that *An. gambiae* s.l. exhibited resistance to bendiocarb (0.1%) and pirimiphos-methyl (1%); and with possible resistance to DDT (4%). IRS was conducted between October and December every year. From 2015, the number of targeted districts were reduced from five to two, Beitbridge and Gwanda. All 15 rural wards in Beitbridge and 8 of 29 rural wards of Gwanda were targeted for IRS. The coverages for rooms sprayed was not consistently universal, as shown Fig. 7.

The IRS programme had adequate protective clothing and equipment. There were two vector sentinel sites in Beitbridge and Matobo Districts. The Matobo District site was not fully functional due to lack of vector mosquito in the district. Long-lasting insecticidal treated nets (LLINs) were last distributed in 2014 in four of the seven districts. Utilization could not be ascertained. Unreliable motorcycles, inadequate fuel, high IRS refusal rate and unavailability of alternative protective methods for the pasturing communities compromised vector control.

### Diagnosis, case management and radial cure

Clinical care was offered across primary, secondary and tertiary levels of care in the province. Nurses were responsible for diagnosis of cases at primary level. They were complimented by Village Health Workers (VHWs) who operate from the community to test and treat malaria cases. Uncomplicated cases were treated with co-artemether upon confirmation with either an RDT or a slide. Complicated cases were treated with parenteral artesunate or quinine with either doxycycline or clindamycin. However, clindamycin was not available. Quinine was more commonly available and more popular than artesunate. Use of primaquine was variable. Primaquine was unavailable prior to November 2015 and Beitbridge District Hospital were still distributing it to clinics.

### Social and behaviour change communication (SBCC)

SBCC activities were carried out throughout the year with special attention to the community level. At rural health centres, nurses and EHTs carry out scheduled and ad-hoc health education sessions to patients. Messages were delivered using posters and pamphlets on utilization of LLINs, seeking early treatment, IRS and malaria elimination. Walls at local shops and branded public transport vehicles with messages on LLINs and IRS were observed. Information and Education Communication material was printed in local languages with messages on malaria elimination. Old, outdated and sometimes irrelevant flip charts which required updating and had malaria control messages were observed.

### Programme management, financing and coordination

All districts of Matabeleland South province were earmarked for halting local malaria transmission by end of 2017. From 2012, malaria elimination has been high on the agenda for the Provincial and District Health Executives. The province developed a malaria elimination strategic plan, annual plans and guidelines on malaria elimination. Six of the seven districts had substantive District Medical Officers for prompt decision making and a Provincial Malaria Focal Person. There was no malaria focal person at district level. There was inadequate funding for preventive services sub vote, which had not received any funding from central government during the period under review. All the procurements for the district were mainly done though coordination with the national level. There were budget lines available for emergency purchases at district level. Both districts had adequate ACT, slides and RDTs at health facility level. However, RDTs were expiring in March 2016. There were shortages of Giemsa stain and glycerol. Primaquine was available at Beitbridge District but had not been rolled out to the health facilities visited. There was no buffer stock of insecticides for IRS. There were no larvicides available.

## Discussion

Matabeleland South province has made considerable progress towards halting local malaria transmission by 2017. Apart from Beitbridge District, all other districts recorded fewer than two cases per 1000 population at risk between 2011 and 2015. Malaria elimination is possible with such low incidence levels. However, Beitbridge District may delay malaria elimination.

Backed by bioassay data, two IRS cycles a year may be justifiable in view of the bimodal nature of malaria transmission in the province. This is even more relevant when using insecticide with much shorter residual action compared to DDT [7]. According to WHO, a “mass” effect is required for IRS to be considered an impactful intervention. This requires that IRS coverage be at least 85% of potential resting spaces sprayed [8]. Despite the low IRS coverages between 2012 and 2014, malaria incidence was below 1 case per 1000 population at risk in all districts except for Beitbridge and Gwanda during the period under review. This suggests that other factors may be responsible for the low incidence in the province. Evidence concerning the concurrent use of LLINs and IRS under conditions of low endemicity is somewhat inconclusive [9]. There is therefore need to explore further reasons for the very low incidence in the districts of Matabeleland South.

The case based surveillance system is similar to the “1-3-7” approach used in China [10]. Unlike the Chinese system, the one in Matabeleland was not robust enough to provide real-time notification of managers via SMS alerts or email notification. This means that if a manager does not log into the DHIS2 system, they may not be aware of recent cases notified in the system. SMS functionality is a crucial component of a robust surveillance system for malaria elimination [11] A similar surveillance system in Swaziland utilizes such notification functions [12]. There is need to managers of the programme in Matabeleland South to review this function of the system.

The burden of proof following malaria elimination lies with the country seeking certification [13]. Matabeleland South province needs to strengthen the accumulation of proof elimination including a foci registers, malaria case registers, investigation forms and annual surveillance reports. Acquiring such documentation presents a strong case for sub-national elimination of malaria and may prove to be good foundation upon which to build on for scale up of elimination activities in Zimbabwe.

Epidemiologically, Beitbridge District is distinct from the other districts in the province. Malaria outbreaks are an important source of local malaria transmission. Whilst local transmission is evident, importation of malaria across the border with South Africa is a potential source of continued malaria transmission. Vhembe District in adjacent South Africa is malaria endemic [14]. There is need to strengthen cross border collaboration with South Africa.

Whilst malaria elimination activities are well established in province, there was little evidence of consistent biolarviciding. Despite its high cost, Larval Source Management is recommended for targeting malaria hotspots to help clear residual foci in elimination settings [15]. The lack of a standardized foci management and response guidance in the province may result in poorly coordinated response especially to foci of different classes.

## Conclusion

Malaria elimination is achievable in Matabeleland South province. This is precedence for sub-national malaria elimination in Zimbabwe. There is need strengthen the case based surveillance with real time notification functionality. Sub-national certification of a malaria free status may assist in gathering elimination evidence and expertise for national scale up. Beitbridge District presents an opportunity for close cross border collaboration.
